# Supplementary material for: The Pro-Oncogenic Protein IF1 Promotes Proliferation of Anoxic Cancer Cells during Re-Oxygenation
Source: Int J Mol Sci. 2023 Sep 27;24(19):14624. doi: 10.3390/ijms241914624 (PMC10572598; doi:10.3390/ijms241914624)
Supplement: Supplementary file 1 [file ijms-24-14624-s001.zip › ijms-2544470-supplementary.pdf]

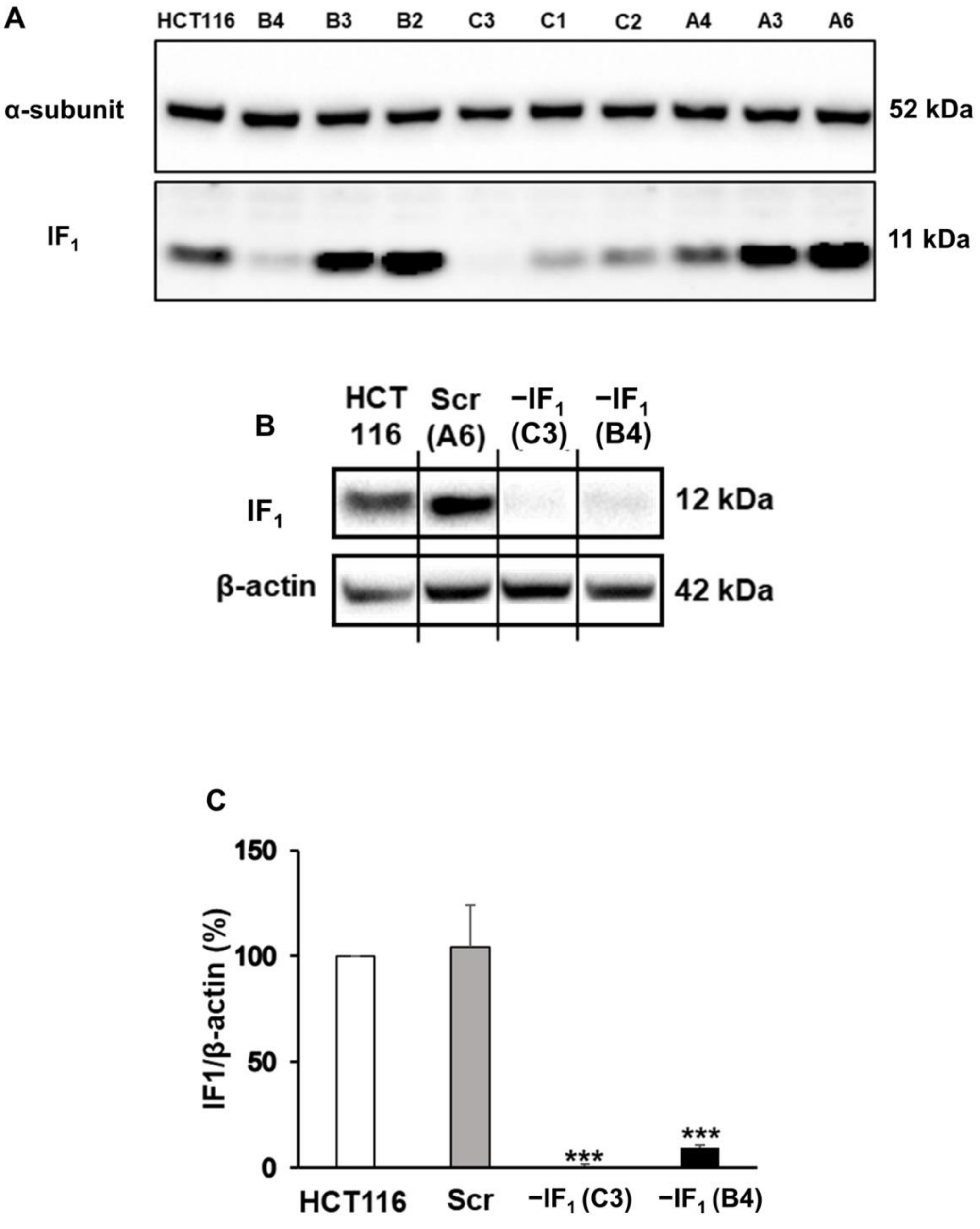

Figure S1: IF$_1$ protein levels in colon carcinoma derived clones and HCT116 parental cell.
(A) Immunodetection of IF$_1$ and ATP synthase **α** subunit protein levels in HCT116 parental cells and derived clones. (B) Immunodetection of IF$_1$ in the colon carcinoma cell types used in the experiments. (C) Densitometric analysis of IF$_1$ normalized to actin and reported as a percentage of the protein content relative to parental cells. Values are means ± SEM ($n = 3$). *** $p < 0.005$ indicates the statistical significance of values compared to parental cells, one simple t test.
